# The Serum Diagnosis of Disease

**Published:** 1900-06

**Authors:** 


THE SERUM DIAGNOSIS OF DISEASE.—By Richard C. Cabot, M. D., Physician to Out-Patients, Massachusetts General Hospital, Boston. 154 pages. New York: William Wood & Co. 1899.

The author is modest enough to say in his prefactory note that "This book is nothing but a compilation." He has done the profession a great service. He has put into convenient form the results of the immense amount of work which has been done upon the serum diagnosis since 1896.

Serum diagnosis marks one of the advances of the last three or four years, and it is explained fully in the above work. It is now possible, by use of the Widal test, to determine whether a given case of fever is typhoid or not. This little book makes it possible for the average practitioner to employ this test in his every day work. It is less difficult than the examination of urinary sediments or for tubercle bacilli. It is to be hoped that this work will introduce serum diagnosis into more general use, and that the practitioner everywhere will fall into line and quit guessing at his fever cases.

